# A Novel Fault Diagnosis Method for a Power Transformer Based on Multi-Scale Approximate Entropy and Optimized Convolutional Networks

**DOI:** 10.3390/e26030186

**Published:** 2024-02-22

**Authors:** Haikun Shang, Zhidong Liu, Yanlei Wei, Shen Zhang

**Affiliations:** Key Laboratory of Modern Power System Simulation and Control & Renewable Energy Technology, Ministry of Education, Northeast Electric Power University, Jilin 132012, China; 2201900107@neepu.edu.cn (Z.L.); 2202200205@neepu.edu.cn (Y.W.); 2202300135@neepu.edu.cn (S.Z.)

**Keywords:** DGA, power transformer, CNNs, sparrow search algorithm, approximate entropy

## Abstract

Dissolved gas analysis (DGA) in transformer oil, which analyzes its gas content, is valuable for promptly detecting potential faults in oil-immersed transformers. Given the limitations of traditional transformer fault diagnostic methods, such as insufficient gas characteristic components and a high misjudgment rate for transformer faults, this study proposes a transformer fault diagnosis model based on multi-scale approximate entropy and optimized convolutional neural networks (CNNs). This study introduces an improved sparrow search algorithm (ISSA) for optimizing CNN parameters, establishing the ISSA-CNN transformer fault diagnosis model. The dissolved gas components in the transformer oil are analyzed, and the multi-scale approximate entropy of the gas content under different fault modes is calculated. The computed entropy values are then used as feature parameters for the ISSA-CNN model to derive diagnostic results. Experimental data analysis demonstrates that multi-scale approximate entropy effectively characterizes the dissolved gas components in the transformer oil, significantly improving the diagnostic efficiency. Comparative analysis with BPNN, ELM, and CNNs validates the effectiveness and superiority of the proposed ISSA-CNN diagnostic model across various evaluation metrics.

## 1. Introduction

Oil-immersed power transformers are vital components in power systems, primarily utilized for voltage regulation and the transmission and distribution of electrical energy [1]. These transformers utilize insulating oil for effective heat control. The design and operation of transformers directly impact the quality of the electrical energy and the reliability of the power system. Therefore, understanding the operational status of oil-immersed transformers and ensuring their safe and stable operation are crucial for the reliability of the power system [2].

The fault diagnosis method for oil-immersed transformers based on dissolved gas analysis (DGA) in oil has gained widespread application in recent years [3,4]. By analyzing the gas content in the transformer oil, this method can effectively identify the types of electrical faults, discover potential issues, and provide crucial information for proactive maintenance of transformers. As a result, it has become increasingly prevalent in the field. Currently, the traditional diagnostic methods for dissolved gases in transformer oil include the three-ratio method [5] and the Duval Triangle method [6]. However, these approaches suffer from shortcomings such as insufficient coding and excessive absoluteness, leading to a higher rate of misjudgment. This phenomenon results in their inability to accurately diagnose certain faults. Therefore, there are now various intelligent diagnostic methods for oil-immersed transformer faults based on DGA. These methods mainly include Artificial Neural Networks (ANNs), Support Vector Machines (SVMs), Expert Systems (ESs), Extreme Learning Machines (ELMs), etc. ANNs offer advantages, such as distributed parallel processing, adaptability, self-learning, associative memory, and non-linear mapping. Zhou et al. [7] proposed a probabilistic neural-network-based fault diagnosis model for power transformers, and the results show that it is applicable to the field of transformer fault diagnosis. However, ANNs suffer from slow convergence and susceptibility to local optima. In paper [8], the multi-layer SVM technique is used to determine the classification of the transformer faults and the name of the dissolved gas. The results demonstrate that combination ratios and the graphical representation technique are more suitable as a gas signature and that an SVM with a Gaussian function outperforms the other kernel functions in its diagnosis accuracy. But SVMs have inherent binary attributes that limit their applications. Mani [9] presented an intuitionistic fuzzy expert system to diagnose several faults in a transformer, and it was successfully used to identify the type of fault developing within a transformer, even if there was conflict in the results when AI techniques were applied to the DGA data. However, ESs rely on rich expert knowledge for diagnosis. Acquiring such knowledge is costly, potentially limiting the diagnostic accuracy. In paper [10], a novel method for transformer fault diagnosis based on a parameter optimization kernel extreme learning machine was proposed. The results verified the effectiveness of the proposed method. ELM features few pre-set parameters, fast training speeds, and suitability for engineering applications. However, it has the drawback of relatively poor learning capabilities. A performance comparison of different diagnostic methods is shown in Table 1.

Data-driven fault detection methods utilize machine learning and data analysis techniques to detect equipment faults [11]. They analyze real-time sensor data or historical data, build models, and compare them with fault patterns. In recent years, these methods have been widely applied in various fields, such as industrial processes [12], HVAC systems [13], energy systems [14], potential fault identification [15], sensor analytics [16], and medical device digital systems [17]. Deep learning theory possesses robust feature learning and pattern recognition capabilities, extracting effective information from large-scale and complex data [18]. In recent years, deep learning, particularly convolutional neural networks (CNNs), has found widespread application in fault diagnosis [19]. CNNs, using convolutional and pooling layers, automatically learn the local and global features from the input data. It can provide effective representation of images, sequences, and so on [20]. The strength of CNNs lies in their efficient processing of complex data and feature learning capabilities. The proper hyperparameters, such as learning rate and filter size, are crucial to the model performance in CNNs. The sparrow search algorithm (SSA) was proposed in 2020 as a novel swarm intelligence optimization algorithm [21]. It primarily achieves position optimization by emulating the foraging and anti-predatory behaviors of sparrows, aiming to locate the local optimum of a given problem [22]. This study introduces an improved sparrow search algorithm (ISSA) for CNN parameter optimization. ISSA can dynamically adjust these parameters to enhance model generalization and robustness. The proposed approach will be applied to transformer fault diagnosis, showcasing the potential of CNNs optimized with ISSA.

The DGA method primarily utilizes the characteristic gas content for transformer fault diagnosis [23]. However, the composition of dissolved gases in oil is highly complex and uncertain. Therefore, assessing the uncertainty solely based on the decomposed gas content is challenging. This study introduces information entropy [24] as a feature indicator for transformer fault diagnosis. Information entropy, a concept from information theory, measures system uncertainty and information quantity. In transformer diagnosis, information entropy can be employed by analyzing the concentration distribution of dissolved gases, assessing system states. Higher entropy values indicate greater system complexity and uncertainty, potentially indicating underlying faults. Information entropy analysis enhances the understanding of system health, supporting early fault detection and prediction [25]. Approximate entropy, a calculation method for information entropy, is commonly used for time-series data analysis [26]. It assesses system complexity and regularity, revealing patterns or trends in data. Multi-scale approximate entropy considers signal characteristics at different scales, observing how complexity evolves with scale changes [27]. This method contributes to a comprehensive understanding of dynamic signal characteristics. It provides in-depth insights into system behavior across different time scales. Currently, approximate entropy has demonstrated effective applications in various fields, including biosignal analysis [28], short-circuiting arc welding analysis [29], mechanical vibration measurements [30], and environmental monitoring [31]. In transformer diagnosis, this paper attempts to enhance early fault prediction by calculating the multi-scale approximate entropy of dissolved gases in oil, offering a more comprehensive insight into system state changes.

This study initially collects the characteristic gas content of oil-immersed transformers under various fault types, including H_2_, CH_4_, C_2_H_6_, C_2_H_4_, and C_2_H_2_. Subsequently, the content ratios of different gas types are obtained. The multi-scale approximate entropy values are then calculated through content ratios to assess the gas complexity. Finally, the multi-scale approximate entropy values serve as feature inputs for an optimized CNN-based classifier, deriving diagnostic results. Field data demonstrate the proposed method’s effectiveness and superiority in transformer fault diagnosis.

The structure of this paper is as follows. The principles of the relevant algorithms are detailed in Section 2. Section 3 presents an oil-immersed transformer fault diagnosis model based on multi-scale approximate entropy and optimized CNNs. Section 4 shows the performance of the proposed diagnostic model. Section 5 concludes the paper.

## 2. Algorithm and Principles

### 2.1. Multi-Scale Approximate Entropy

#### 2.1.1. Approximate Entropy

Approximate entropy is a non-linear dynamical parameter used to quantify the regularity and unpredictability of fluctuations in a time series. It is represented by a non-negative number that reflects the complexity of a time series, indicating the likelihood of new information occurring in the time series. The more complex the time series, the higher the corresponding approximate entropy.

#### 2.1.2. Algorithm Steps

Let the original signal be a time series containing *N* data points *u*(1), *u*(2), *u*(3),…, *u*(*N*);

Generate a set of vectors with a dimension of *m*: *x*(1), *x*(2), *x*(3),…, *x*(*N* − *m* + 1), where *m* represents the length of the window.

(1) x(i)={u(i),u(i+1),⋯,u(i+m−1)}, i∈[1,N−m+1]

Define the distance between *x*(*i*) and *x*(*j*) as *d*[*x*(*i*), *x*(*j*)] to be the maximum of the absolute differences between their corresponding elements.

(2) $$d[x(i),x(j)]=\max[\left|x(i+k)-x(j+k)\right|]$$

Given a threshold *r*, for each value of *i*, count the number of distances *d* that are less than *r*, and calculate the ratio of this count to the total number of distances *N* − *m*.

(3) $$C_{i}^{m}(r)=\frac{1}{N-m}\{d[x(i),x(j)]<r\}$$

Take the logarithm of $C_{i}^{m}(r)$, and then calculate the average across all *i* in Equation (4).

(4) $$\phi^{m}(r)=\frac{1}{N-m+1}\sum_{i=1}^{N-m+1}\ln C_{i}^{m}(r)$$

Increase the dimension by 1 to *m* + 1, then repeat steps (2) to (5), resulting in $C_{i}^{m+1}(r)$ and $\phi^{m+1}(r)$.

In theory, the approximate entropy of this sequence is defined as:

(5) $$ApEn(m,r)=\underset{N\to \infty }{\lim}[\phi^{m}(r)-\phi^{m+1}(r)]$$

When *N* is a finite value, the *ApEn* estimate obtained by following the above steps for a sequence of length *N* is denoted as:

(6) $$ApEn(m,r,N)=\phi^{m}(r)-\phi^{m+1}(r)$$

#### 2.1.3. Multi-Scale Approximate Entropy

Multi-scale approximate entropy (MApEn) extends the concept of approximate entropy to multiple time scales. It provides additional perspectives when dealing with data of uncertain time scales. The approximate entropy does not adequately account for different time scales that may exist within a time series. The objective of multi-scale entropy is to assess the complexity of time series.

The fundamental principle of multi-scale entropy involves coarsening or downsampling, primarily analyzing the time series at progressively coarser time resolutions. Coarse-grained data take the average of different numbers of consecutive data points to create signals at different scales. The specific steps are as follows.

When Scale = 1, the coarse-grained data are the original time series.

When Scale = 2, the coarse-grained time series is formed by calculating the average of two consecutive time points, as defined in Equations (7) and (8).

(7) $$y_{1,j}^{\left(2\right)}=\frac{x_{i}+x_{i+1}}{2}$$

(8) $$y_{2,j}^{\left(2\right)}=\frac{x_{i+1}+x_{i+2}}{2}$$

**Figure 1:**
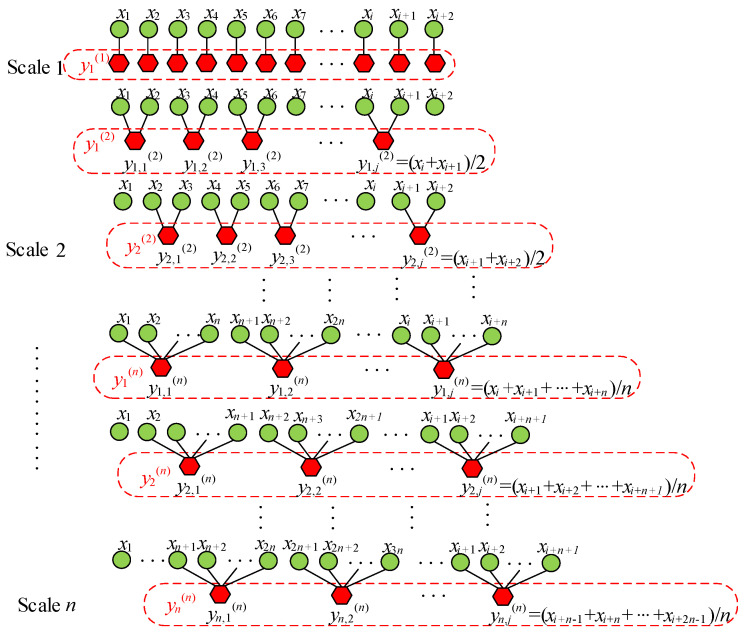
Schematic of the coarse-grained process.

Similarly, when Scale = *n*, the coarse-grained time series is formed by taking the average of n consecutive time points, as shown in Figure 1.

The mathematical definition of the above coarse-grained process is as follows.

(9) $$y_{j}^{\left(\tau \right)}=\frac{1}{\tau }\sum_{i=(j-1)\tau +1}^{j\tau }X_{i},\;1\leq j\leq \frac{N}{\tau }$$

where *τ* represents the time scale.

### 2.2. Improved Sparrow Search Algorithm

SSA is a heuristic optimization method inspired by the collective behaviors of sparrow bird populations. It utilizes a combination of individual exploration and information-sharing strategies to address optimization challenges. This algorithm is conceived as an optimization approach that draws inspiration from the foraging and migration patterns of sparrows. However, the SSA algorithm is susceptible to the influence of problem complexity and parameter settings, resulting in slow convergence and low accuracy. In this article, the following improvement strategies are proposed.

(1) This article employs chaotic mapping for the initialization of the SSA population to achieve stable population quality. The generated chaotic sequences are as described in Equation (10).

(10) $$Z_{I+1}^{K}=\left\{\begin{array}{l} \frac{Z_{I}^{K}}{u},\;0\leq Z_{I}^{K}\leq u\\ \frac{1-Z_{I}^{K}}{1-u},\;u\leq Z_{I}^{K}\leq 1 \end{array}\right.$$

In this context, where *K* represents the population size and *I* is the current iteration count, *u* takes on random values between 0 and 1. The process for initial position generation of sparrow individuals using the chaotic sequence is as follows.

(11) $$X_{I}^{K}=(\min)X_{I}^{K}+Z_{I}^{K}*((\max)X_{I}^{K}-(\min)X_{I}^{K})$$

where $(\min)X_{I}^{K}$ and $(\max)X_{I}^{K}$ represent the minimum and maximum values of $X_{I}^{K}$, respectively.

(2) To prevent being stuck in local optima, this article introduces a non-linearly decreasing weight $\omega_{m}$ in the update of SSA discoverer positions. The calculation formula is as follows.

(12) $$\omega_{m}=\omega_{1}(\omega_{1}-\omega_{2})(1-\tan\frac{\pi t}{4t_{\max}}\frac{t^{2}}{t_{\max}^{2}})$$

where *ω*_1_ and *ω*_2_ are inertia adjustment parameters with values of *ω*_1_ = 0.9 and *ω*_2_ = 0.4, and *t*_max_ represents the maximum number of iterations. The weight has a slower decay at the beginning of iterations, favoring global search for the optimal solution’s position. 

(3) This article introduces a mutation strategy to update the contributors. A Gaussian mutation operator is introduced to perturb the global best solution. This can prevent being trapped in local optima. Gaussian mutation operator is defined in Equation (13).

(13) $$X_{gauss}^{t+1}=X_{gauss}^{t}(1+Gaussian(\alpha ))$$

where $X_{gauss}^{t+1}$ represents the Gaussian best solution, and Gaussian(α) denotes a random vector following a Gaussian distribution, with a mean of 0 and a variance of 1.

**Figure 2:**
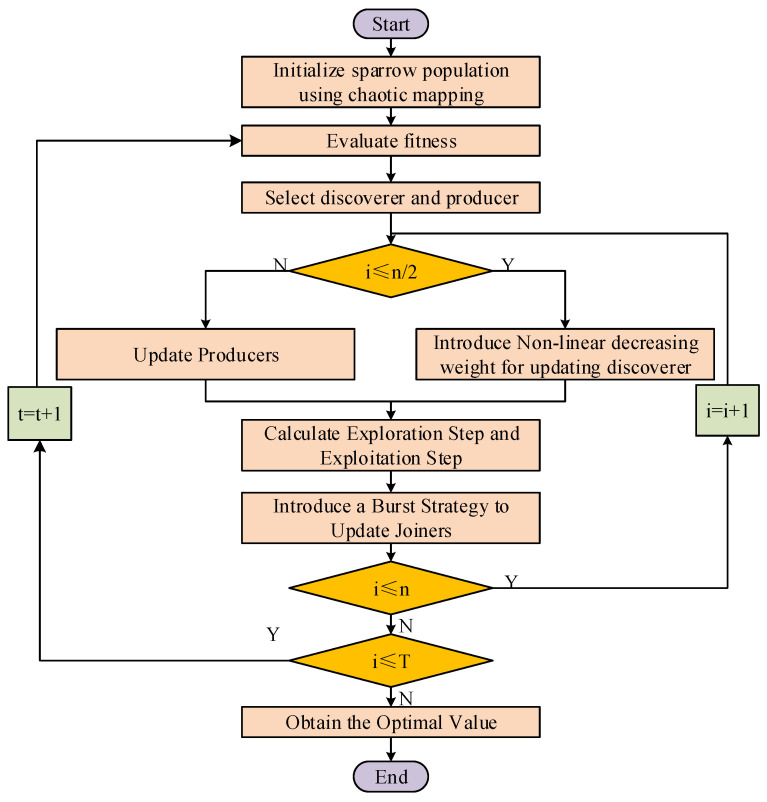
The flowchart of ISSA.

A flowchart of the ISSA is shown in Figure 2.

**Figure 3:**
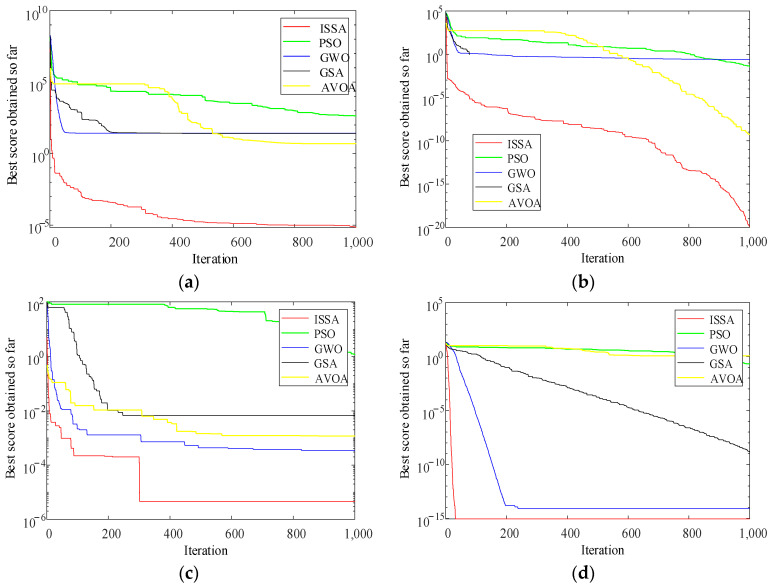

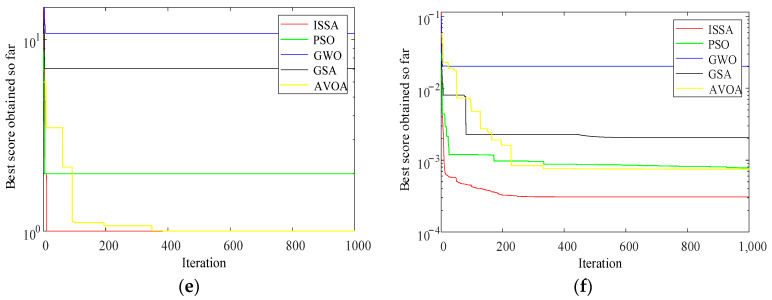
Fitness curves of different algorithms. (**a**) F1; (**b**) F2; (**c**) F3; (**d**) F4; (**e**) F5; (**f**) F6.

To validate the performance of ISSA, this study conducted numerical simulation experiments on six selected functions from the Congress on Evolutionary Computation test suite [32]. A comparative analysis was carried out with Particle Swarm Optimization (PSO) [33], Grey Wolf Optimizer (GWO) [34], Gravitational Search Algorithm (GSA) [35], and African Vultures Optimization Algorithm (AVOA) [36]. The six functions and their parameters are listed in Table 2. The convergence curves of different algorithms are illustrated in Figure 3.

From Figure 3, it can be observed that ISSA exhibits the fastest convergence speed on various test functions, demonstrating significantly better performance compared to PSO, GSA, GWO, and AVOA.

### 2.3. CNNs

CNNs are a type of deep feedforward neural network with a hierarchical structure. The architecture primarily includes convolutional layers, pooling layers, activation layers, and fully connected layers.

#### 2.3.1. Convolutional Layer

The main role of the convolutional layer in CNNs is to perform feature extraction on the input. The convolutional kernels in different layers have varying sizes, allowing the network to capture features of different scales. As a result, CNNs can extract multi-scale feature information. The calculation formula for the output value $a_{j}^{l}$ of the *j*th unit in convolutional layer *l* is as follows.

(14) $$a_{j}^{l}=f(b_{j}^{l}+\sum_{i\in M_{j}^{l}}a_{j}^{l-1}*k_{ij}^{l})$$

where $M_{j}^{l}$ represents the selected set of input feature maps, and *k* represents the learnable convolutional kernel.

#### 2.3.2. Pooling Layer

Pooling operations are performed independently on each subset of data.. The purpose of pooling is to gradually reduce the spatial dimensions of the data volume. This helps reduce the number of parameters in the network, saving computational resources effectively. Pooling is commonly used during upsampling and downsampling processes. It has no learnable parameters. The calculation of activation value in pooling layer *l* is based on Equation (15).

(15) $$a_{j}^{l}=f(b_{j}^{l}+\beta_{j}^{l-1}down(a_{j}^{l-1},M^{l}))$$

where *down*(.) represents pooling function, $b_{j}^{l}$ is the bias, $\beta_{j}^{l}$ means the multiplicative residual, and *M*^l^ represents the size of the pooling window.

#### 2.3.3. Activation Layer

CNNs are composed of multiple layers of composite functions. Rectified Linear Unit (ReLU) is a widely used activation function in CNNs. Its sparse representation can accelerate learning and simplify models. The mathematical expression of the ReLU function is as follows.

(16) f(p)=max(0,p)

For an input *p*, the ReLU function returns an output equal to the maximum value between *p* and 0. If *p* is greater than or equal to 0, the output is *p* itself; otherwise, the output is 0.

#### 2.3.4. Fully Connected Layer

The parameters in the fully connected layer include the total number of fully connected layers and the number of neurons in each individual layer. Increasing the width of the fully connected layer and the number of layers can enhance the model’s non-linear expressive power.

### 2.4. Optimized CNNs with ISSA

**Figure 4:**
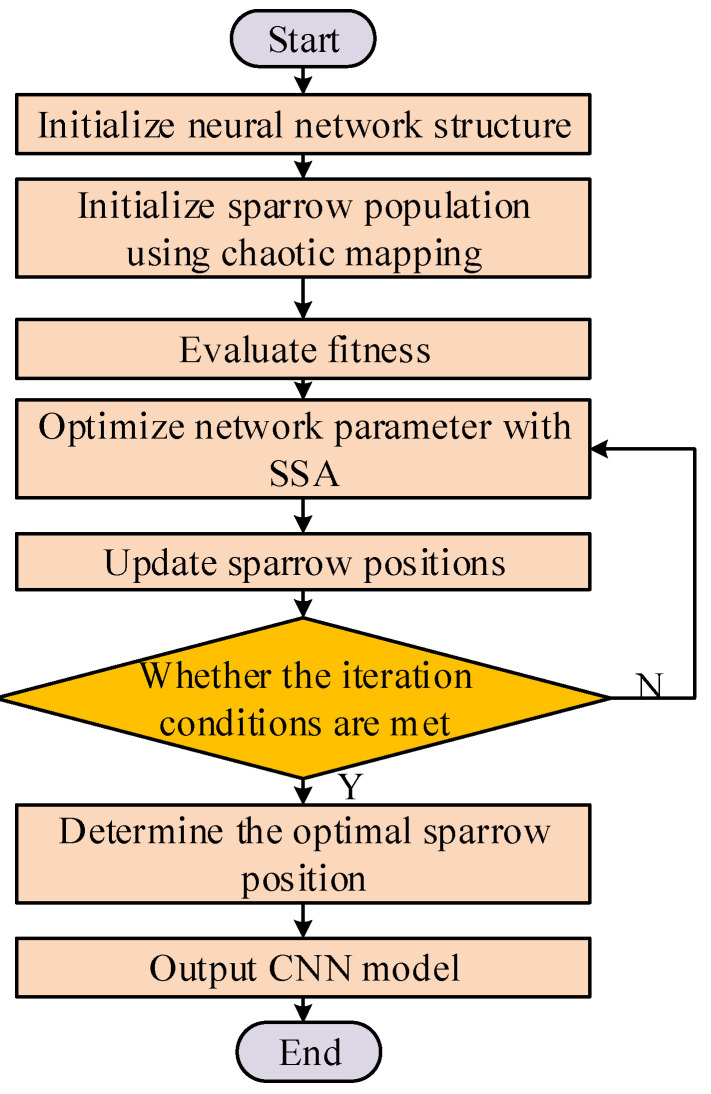
The process of CNNs based on ISSA.

The basic process of CNNs based on ISSA is illustrated in Figure 4.

## 3. Power Transformer Fault Diagnosis Based on Multi-Scale Approximate Entropy and Optimized Deep Convolutional Networks

**Figure 5:**
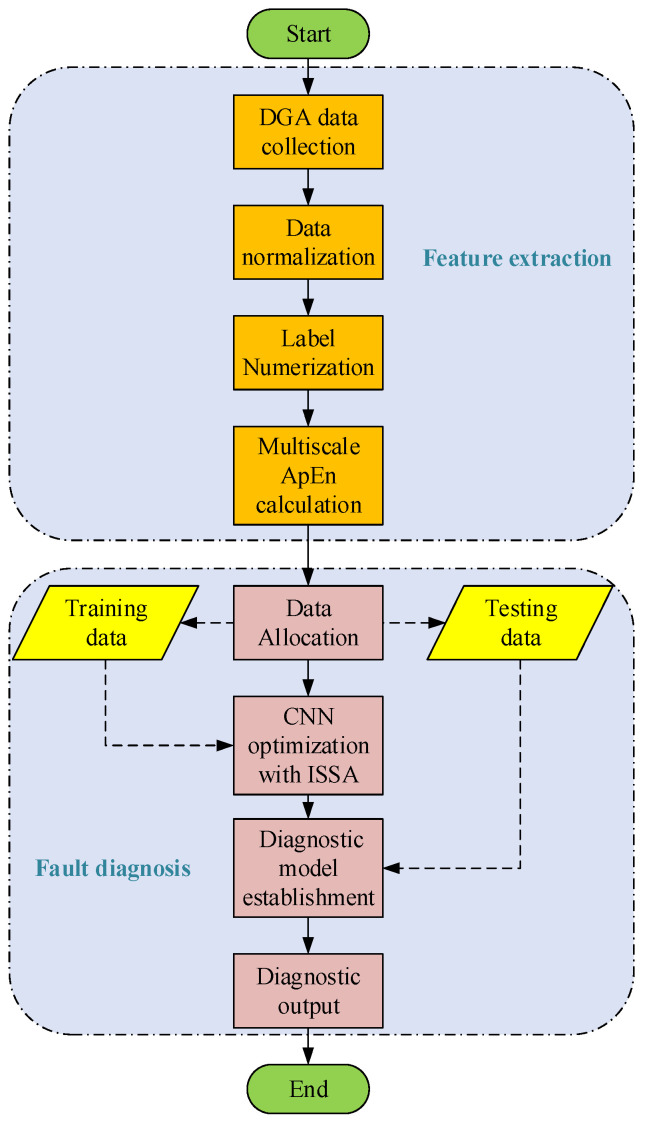
Power transformer fault diagnosis procedure.

This study utilizes the optimized convolutional neural network for the analysis of dissolved gases in transformer oil. Initially, eight types of dissolved gases in transformer oil are collected. Subsequently, the gas contents are numerically labeled and normalized. The multi-scale approximate entropy is employed for feature extraction on the pre-processed data. Finally, the extracted features are fed into the optimized convolutional neural network for fault diagnosis. The diagnostic process is illustrated in Figure 5.

## 4. Case Study Analysis

### 4.1. Data Preprocessing

The raw data used in this study consist of actual measurements of dissolved gases in transformer oil from a certain substation, totaling 555 sets. Some of the transformer parameters are present in Table 3.

Each set of data includes five features along with the corresponding eight data types, including normal type (NT), high-energy discharge (HD), low-energy discharge (LD), high-temperature overheating (HO), intermediate-temperature overheating (ITO), intermediate- to low-temperature overheating (ILO), low-temperature overheating (LO), and partial discharge (PD). Some of the gas chromatography data are presented in Table 4.

Due to significant differences in gas content values corresponding to different fault types, this study performs standardization on the gas contents using Equation (17). The processed data are presented in Table 5.

(17) $$x^{\prime }=\frac{x-\min(x)}{\max(x)-\min(x)}$$

Due to the correlation between transformer fault types and the corresponding gas content ratios, gas ratios are commonly used as input data in transformer fault diagnosis. In this work, 21 gas ratios were obtained, as shown in Table 6.

In Table 6, ALL = CH_4_ + C_2_H_6_ + C_2_H_4_ + C_2_H_2_ + H_2_, TD = CH_4_ + C_2_H_4_ + C_2_H_2_, TH = CH_4_ + C_2_H_6_ + C_2_H_4_ + C_2_H_2_. Partial gas ratio data obtained after calculation are shown in Table 7.

### 4.2. Feature Extraction

**Figure 6:**
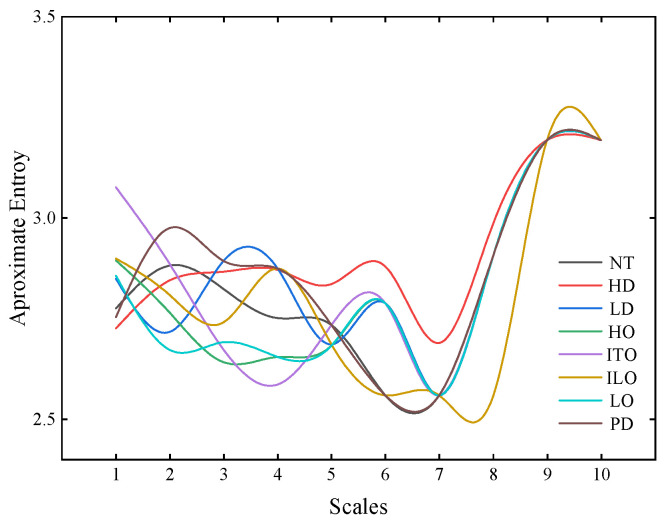
Approximate entropy values varying with different scales.

To extract valuable feature information from the aforementioned gas ratios, multi-scale approximate entropy is introduced to extract characteristic parameters from the gas ratio data. With an approximate entropy scale set to 10, the obtained approximate entropy values for different types of gas contents are illustrated in Figure 6.

Figure 6 shows that when the scale is greater than 6, the approximate entropy values for different transformer faults are relatively similar, exhibiting the same changing trend. At scale values of 4, 5, and 6, there still exist some fault types with similar approximate entropy values. However, at a scale value of 3, the differences in approximate entropy values among different faults become more distinct. Considering that a low scale may lead to the loss of sample information, the scale is set to 3 in this study.

**Figure 7:**
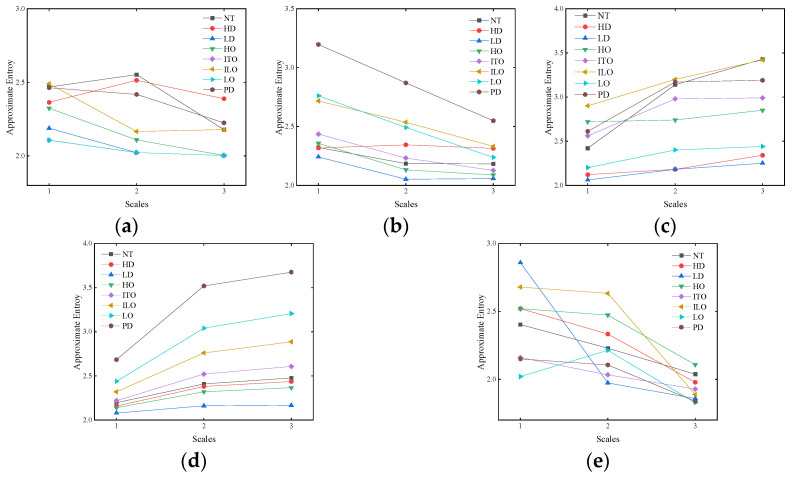
Comparison with different embedding dimensions. (**a**) m = 2; (**b**) m = 3; (**c**) m = 4; (**d**) m = 5; (**e**) m = 6.

Taking into account the impact of different embedding dimensions on entropy values, the embedding dimensions range from 2 to 6. Figure 7 presents the comparative results for different fault types under different embedding dimensions.

The impact of the embedding dimension for different fault types is evident from Figure 7. When *m* takes values of 2, 3, 4, and 6, there is a drastic fluctuation in approximate entropy values, leading to potential confusion between different fault types. However, when *m* is set to 5, the approximate entropy values for different fault types exhibit a more gradual change with increasing scales. Therefore, *m* is set to 5. Partial results are presented in Table 8.

### 4.3. Optimized CNNs with ISSA

This section utilizes the extracted data to train a CNN model. Initially, the ISSA optimization method is employed to fine-tune the CNN hyperparameters, with a maximum training iteration set to 10. The discoverer’s proportion in the population is determined to be 20%. The parameters are presented in Table 9.

**Figure 8:**
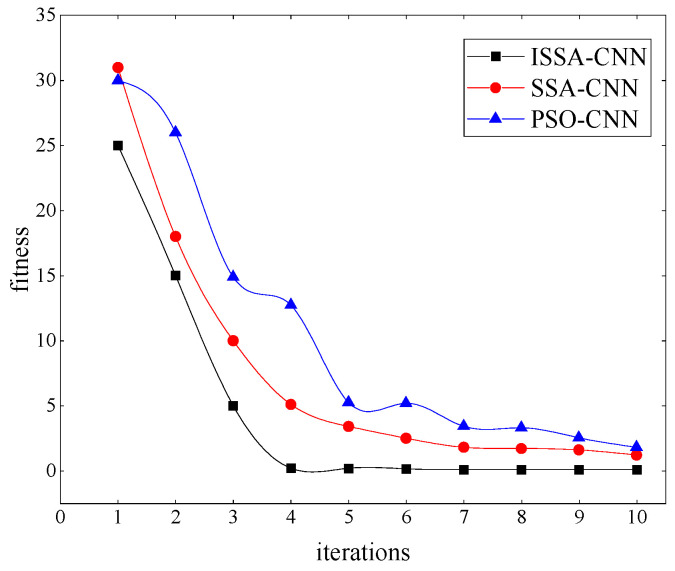
Fitness curve of different optimization methods.

Figure 8 depicts the fitness curves obtained through testing with PSO-CNN, SSA-CNN, and ISSA-CNN, respectively.

From Figure 8, it is evident that, compared to PSO-CNN and SSA-CNN, ISSA-CNN converges more rapidly to a stable fitness value, indicating its superior optimization effectiveness.

### 4.4. Results Analysis

**Figure 9:**
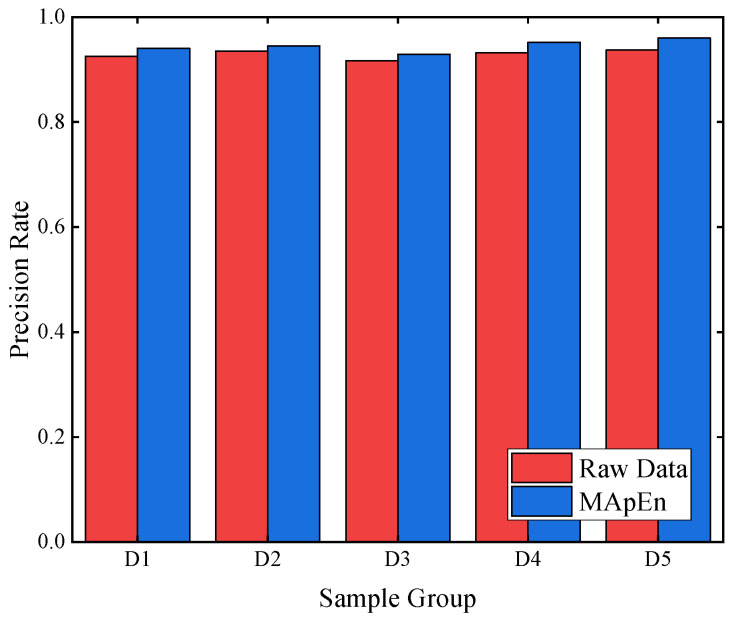
Test results.

**Figure 10:**
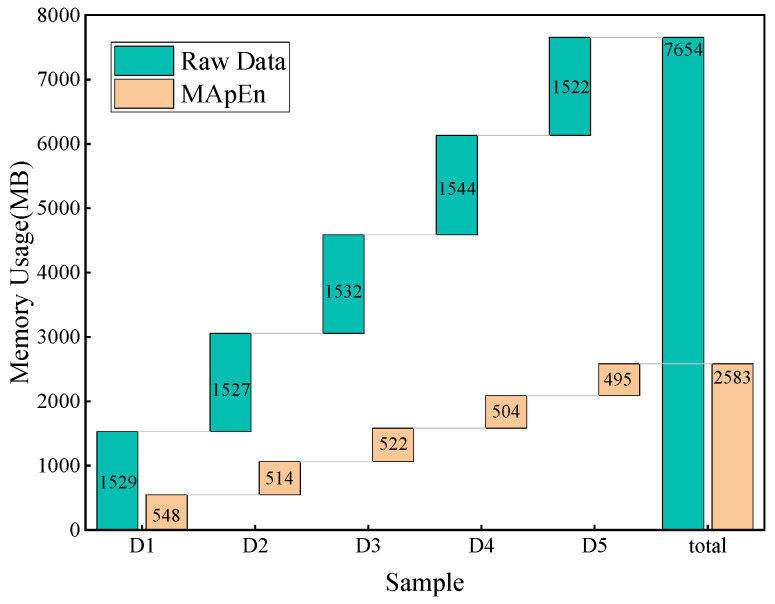
The memory consumption before and after feature extraction.

**Figure 11:**
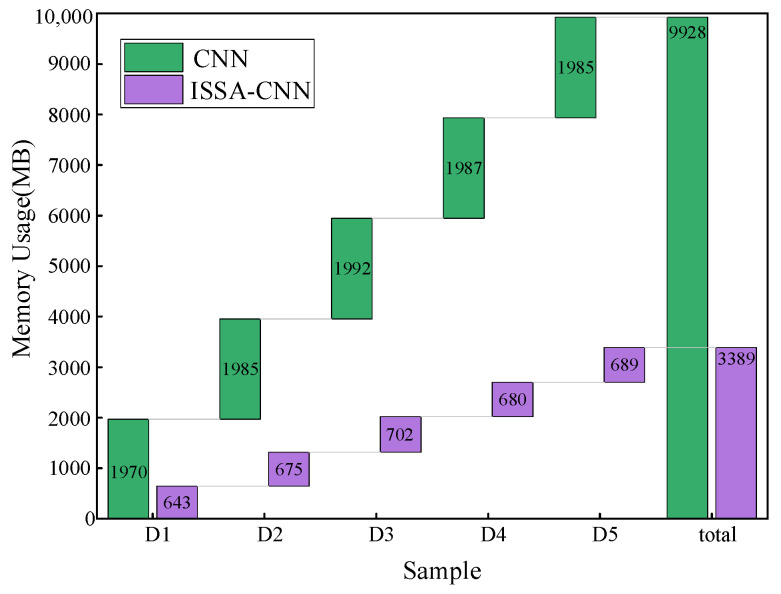
The memory consumption before and after optimization via ISSA.

This study analyzes 555 sets of transformer data, comparing the situations before and after feature extraction. Five-fold cross-validation is employed in this study, where the sample data are randomly divided into five equal parts, namely D1, D2, D3, D4, and D5. Each part is used as a test set in turn, while the remaining four parts serve as training sets. The testing results are illustrated in Figure 9. Raw data represent the original data of 21 gas ratios, while MApEn denotes the multi-scale approximate entropy values. The memory consumption before and after feature extraction is presented in Figure 10. The memory consumption of the model before and after optimization with ISSA is presented in Figure 11.

Figure 9 indicates that after utilizing multi-scale approximate entropy for feature extraction in transformer data, the diagnostic results for different partitions show better performance compared to the diagnostic performance before feature extraction. It indicates that the feature extraction method in this study can collect valuable transformer data information and eliminate easily confused redundant information.

Figure 10 shows that the memory consumption of the model in processing data is much lower after feature extraction compared to that before feature extraction. This indicates that the feature extraction method in this study significantly improves the efficiency of diagnostic operations.

Figure 11 indicates that the memory consumption of the model during fault diagnosis is much lower after ISSA optimization. This means that the ISSA method significantly improves the efficiency of diagnostic operations.

**Figure 12:**
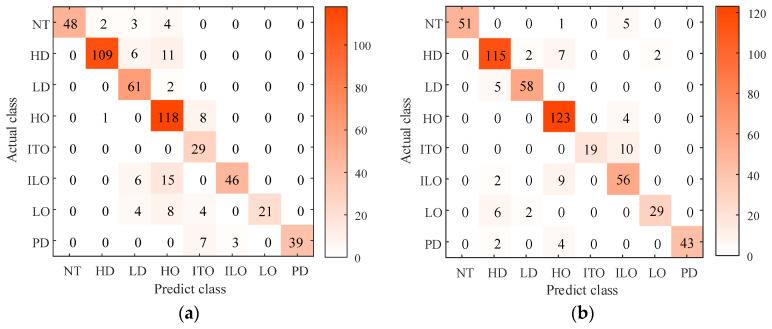

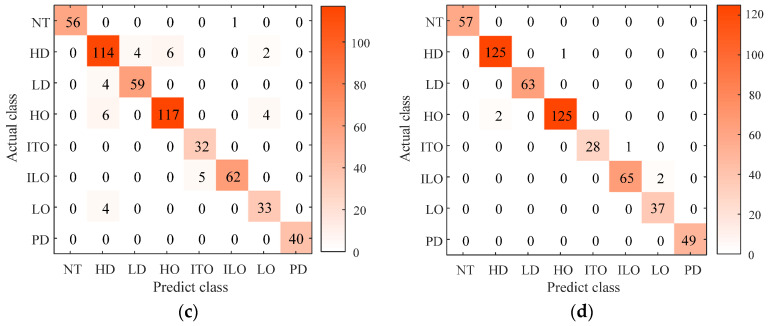
Diagnostic accuracy of different algorithms. (**a**) BPNN; (**b**) ELM; (**c**) CNN; (**d**) ISSA-CNN.

In order to thoroughly validate the superiority of the proposed transformer fault diagnostic model, three algorithms, including BPNN, ELM, and CNN, are introduced in this study for comparative analysis. The confusion matrix obtained through a five-fold cross-validation method is presented in Figure 12.

In Figure 12, it is evident that different diagnostic methods yield significantly different diagnostic results. As seen in Figure 12a, the diagnostic performance of the BPNN method is relatively poor. Although it accurately identifies data with ITO, it struggles to recognize other types of transformer faults. In Figure 12b, the ELM method improves the diagnostic accuracy but still exhibits noticeable misjudgments, making it difficult to differentiate between ITO and ILO. The results in Figure 12c indicate that the CNN, compared to the first two algorithms, achieves an overall improvement in recognition accuracy. However, there are still clear misjudgments in identifying fault labels. In Figure 12d, it can be observed that the CNN classification model optimized through ISSA demonstrates excellent recognition performance, meeting the engineering requirements.

To provide a comprehensive assessment of the proposed model’s performance, this paper employs accuracy, precision, recall, F1-score, and Kappa coefficient for analysis. Accuracy is a fundamental metric for evaluating the performance of a classification model, measuring the ratio of correctly classified samples to the total number of samples. Precision represents the proportion of true-positive samples among those predicted as positive. Recall indicates the ratio of samples predicted as positive to all actual positive samples. F1-score is a metric that combines precision and recall, representing their harmonic mean. The Kappa coefficient is a statistical measure of classification model performance, considering the difference between the model’s performance and random classification. The Kappa coefficient value ranges from −1 to 1, where 1 signifies perfect agreement, 0 indicates no difference from random classification, and −1 denotes complete disagreement. The calculation method is as shown in Equations (18)–(23).

(18) $$Accuracy=\frac{TP+TN}{TP+TN+FP+FN}$$

(19) $$Precision=\frac{TP}{TP+FP}$$

(20) $$Recall=\frac{TP}{TP+FN}$$

(21) $$F1-score=\frac{2*Precision*Recall}{Precision+Recall}$$

(22) $$Kappa=\frac{Accuracy-P(E)}{1-P(E)}$$

where *TP* represents true positive, *TN* represents true negative, *FP* represents false positive, and *FN* represents false negative. *P*(*E*) is the expected accuracy, calculated in Equation (23), representing the performance under random conditions.

(23) $$P(E)=\frac{\left(TP+FP)*(TP+FN)+(TN+FN)*(TN+FP\right)}{{\left(TP+TN+FP+FN\right)}^{2}}$$

**Figure 13:**
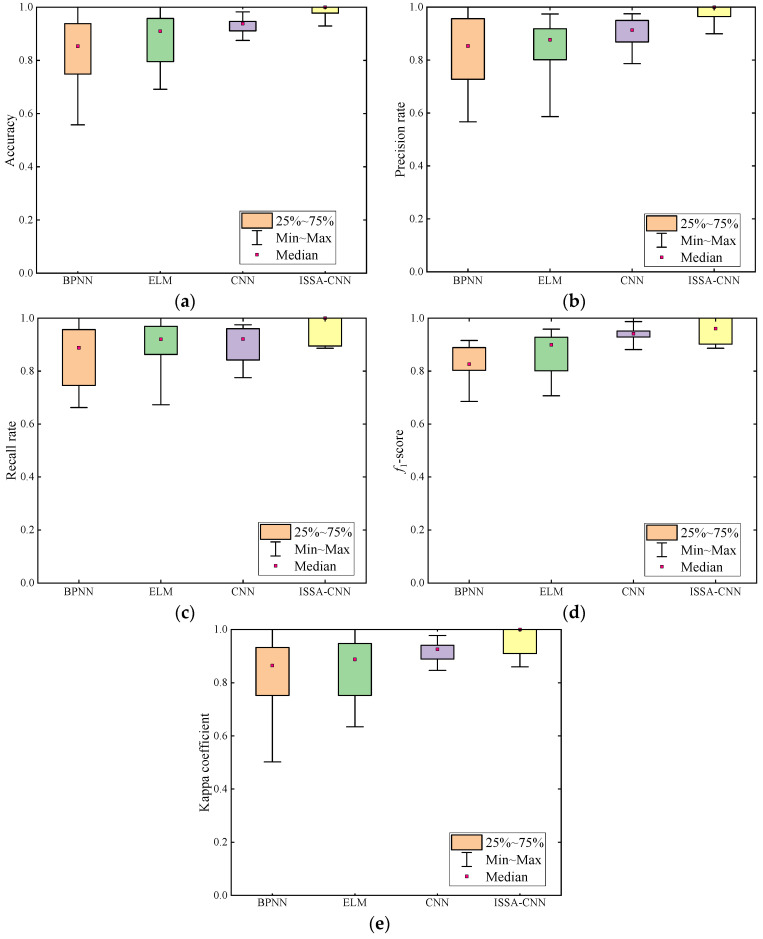
Different methods of assessing results and analysis. (**a**) Accuracy; (**b**) precision rate; (**c**) recall rate; (**d**) F1-score; (**e**) Kappa coefficient.

The diagnostic results for different methods are illustrated in Figure 13. The box in the figure represents the interquartile range from the upper quartile to the lower quartile. The upper and lower whiskers, respectively, depict the maximum and minimum values. The median point represents the middle value, indicating the average level of the metrics calculated by this method.

Figure 13a reveals significant differences in fault accuracy among the four diagnostic methods, and the ISSA-CNN method exhibits a distinct advantage compared to the other three methods. The diagnostic results in Figure 13b indicate that ISSA-CNN achieves higher precision in terms of maximum, minimum, and average values, demonstrating superior recognition performance within the limited transformer data range. The results in Figure 13c suggest that the recall rate of the proposed method is higher, indicating a greater number of correctly predicted samples and a clear advantage in diagnostic effectiveness. As shown in Figure 13d, the F1-scores obtained by ISSA-CNN are distributed above 85%, indicating excellent generalization performance. Figure 13e demonstrates that the Kappa coefficient of ISSA-CNN has a minimum value and overlapping boxes, indicating a stable distribution range and high classification accuracy.

## 5. Conclusions

Building upon the analysis of dissolved gases in transformer oil, this study proposes the ISSA-CNN model for transformer fault diagnosis. The conclusions are as follows.

1. This study introduces an improved sparrow search algorithm that incorporates enhancement strategies in population initialization and position updating. The effectiveness of the enhanced algorithm is validated through optimizing test functions. The algorithm is then applied to optimize the hyperparameters of CNNs. Comparative analysis with different optimization algorithms and validation on the DGA dataset demonstrates its superiority.
2. This study analyzes eight different types of transformer oil and gas data, deriving 21 gas ratios. Subsequently, multi-scale approximate entropy is calculated for these gas ratio contents. The uncertainty of dissolved gases in transformer oil is represented by entropy values, and the multi-scale approximate entropy values are used as feature vectors input into the optimized CNN diagnostic model. The results indicate that the extracted multi-scale approximate entropy can effectively characterize dissolved gas contents and improve the diagnostic effectiveness.
3. To verify the effectiveness and superiority of the proposed method, this study compares it with BPNN, ELM, and CNNs. The results show that the ISSA-CNN transformer fault diagnosis model outperforms the other three methods in terms of accuracy, recall rate, precision, F1-score, and Kappa coefficient. This indicates that the proposed method has good generalization performance and demonstrates favorable application effects in transformer fault diagnosis.

In the future: the authors will attempt to collect more on-site transformer fault data to validate the effectiveness and practicality of the proposed model. Additionally, further improvements can be made to better optimize the parameters of the convolutional neural network and enhance the robustness and stability of the model.

## Figures and Tables

**Table 1:** Comparison of different diagnostic methods.

Methods		Advantages	Disadvantages
Traditional diagnostic methods	the three-ratio method	Easy to understand, rich in experience	Low accuracy, high reliance on experience
	the Duval Triangle method	Intuitive presentation, comprehensive consideration	Subjectivity, reliance on experience
Intelligent diagnostic methods	ANNs	Strong learning ability, fast calculation speed	Slow training, difficult interpretation
	SVMs	Good generalization ability, strong computational power	Sensitive to parameter selection, difficult to interpret results
	ES	Consistent decision provision, expert knowledge storage	Low adaptability, poor interpretability
	ELM	Fast training speed, efficient memory usage	Sensitive to parameters and outliers

**Table 2:** Test function parameters.

Test Functions	Search Range	Optimal Value	Dimension
$F_{1}(x)=\sum_{i=1}^{n-1}\left[100(x_{i+1}-x_{i}^{2})+{\left(x_{i}-1\right)}^{2}\right]$	[−30, 30]	0	30
$F_{2}(x)=\sum_{i=1}^{n}{\left(\left[x_{i}+0.5\right]\right)}^{2}$	[−100, 100]	0	30
$F_{3}(x)=\sum_{i=1}^{n}ix_{i}^{4}+random[0,1)$	[−1.28, 1.28]	0	30
$F_{4}(x)=-20\exp\left(-0.2\sqrt{\frac{1}{n}\sum_{i=1}^{n}x_{i}^{2}}\right)-\exp\left(\frac{1}{n}\sum_{i=1}^{n}\cos\left(2\pi x_{i}\right)\right)+20+e$	[−32, 32]	0	30
$F_{5}(x)={\left(\frac{1}{500}+\sum_{j=1}^{25}\frac{1}{j+\sum_{i=1}^{2}{\left(x_{i}-a_{ij}\right)}^{6}}\right)}^{-1}$	[−65, 65]	1	2
$F_{6}(x)={\sum_{i=1}^{11}\left[a_{i}-\frac{x_{1}(b_{i}^{2}+b_{1}x_{2})}{b_{i}^{2}+b_{1}x_{3}+x_{4}}\right]}^{2}$	[−5, 5]	0.000387	4

**Table 3:** Transformer parameters.

Type	Parameters
Substation Voltage Level	750 kV
Transformer Model	ODFPS-500000/750
Production Time	June 2018
Commissioning Date	14 May 2019
Main Transformer Cooler Model	YF-400

**Table 4:** Transformer oil chromatography data.

Sequence	H_2_	CH_4_	C_2_H_6_	C_2_H_4_	C_2_H_2_	Data Type
1	0.00	1.78	1.02	2.16	0.42	NT
2	130.00	98.00	7.00	56.00	65.00	HD
3	428.00	1660.00	533.00	4094.00	11.40	HO
4	97.81	15.87	2.71	8.10	24.36	LD
…	…	…	…	…	…	…
555	17.30	3.20	6.50	4.50	0.40	PD

**Table 5:** Normalized results.

Sequence	H_2_	CH_4_	C_2_H_6_	C_2_H_4_	C_2_H_2_	Data Type
1	0.000	0.824	0.472	1.000	0.194	NT
2	1.000	0.750	0.039	0.422	0.492	HD
3	0.104	0.405	0.129	1.000	0.002	HO
4	1.000	0.138	0.000	0.057	0.228	LD
…	…	…	…	…	…	…
555	1.000	0.166	0.361	0.243	0.000	PD

**Table 6:** 21 Gas ratios.

Index	Gas Ratios	Index	Gas Ratios	Index	Gas Ratios
1	CH_4_/H_2_	8	C_2_H_2_/C_2_H_4_	15	C_2_H_6_/C_2_H_4_
2	C_2_H_4_/H_2_	9	H_2_/TH	16	CH_4_/TD
3	C_2_H_6_/ALL	10	C_2_H_6_/H_2_	17	C_2_H_2_/TD
4	C_2_H_6_/CH_4_	11	C_2_H_6_/TH	18	C_2_H_2_/CH_4_
5	H_2_/ALL	12	C_2_H_4_/ALL	19	C_2_H_2_/H_2_
6	C_2_H_2_/C_2_H_6_	13	CH_4_/TH	20	C_2_H_2_/TH
7	C_2_H_4_/TD	14	CH_4_/ALL	21	C_2_H_2_/ALL

**Table 7:** Partial gas ratio.

1	2	3	4	5	6	7	8	9	10	11	12	13	14	15	16	17	18	19	20	21	Data Type
178	102	42	0.2	0.2	0.4	0.5	0.6	0.3	0.4	0.2	0.1	0.0	0.1	0.5	0.4	0.0	0.3	0.2	178	102	NT
0.8	0.1	0.5	1.2	0.7	9.3	0.1	0.1	0.4	0.2	0.0	0.3	0.6	0.3	0.3	0.4	0.4	0.3	0.0	0.8	0.1	HD
3.9	1.2	0.0	0.0	0.0	0.0	0.1	0.3	0.3	0.7	0.1	0.0	0.1	0.0	0.7	0.3	0.1	0.2	0.1	3.9	1.2	HO
0.2	0.0	0.2	3.0	1.5	9.0	0.3	0.2	0.3	0.2	0.1	0.5	1.9	0.5	0.2	0.3	0.7	0.1	0.0	0.2	0.0	LD
1.3	0.2	0.0	0.0	0.0	0.0	0.2	0.1	0.6	0.4	0.1	0.0	0.4	0.0	0.4	0.6	0.3	0.4	0.0	1.3	0.2	LO
1.7	0.4	0.0	0.0	0.0	0.0	0.2	0.2	0.4	0.5	0.1	0.0	0.2	0.0	0.6	0.4	0.2	0.3	0.1	1.7	0.4	ITO
1.2	1.0	0.0	0.0	0.0	0.0	0.8	0.9	0.3	0.4	0.3	0.0	0.3	0.0	0.5	0.5	0.2	0.3	0.2	1.2	1.0	ILO
…	…	…	…	…	…	…	…	…	…	…	…	…	…	…	…	…	…	…	…	…	…
0.2	0.4	0.0	0.1	0.1	0.1	1.4	2.0	0.2	0.3	0.4	0.0	1.2	0.0	0.6	0.4	0.5	0.1	0.2	0.1	0.0	PD

**Table 8:** Partial multi-scale entropy value results.

Scale 1	Scale 2	Scale 3	Data Type
3.41	2.85	2.67	HO
3.36	2.8	2.35	LD
2.71	2.86	2.96	HO
3.40	2.89	2.59	ILO
3.40	2.98	2.69	PD
3.34	3.07	2.96	PD
…	…	…	…
3.44	3.11	3.04	HD

**Table 9:** CNN hyperparameters.

Hyperparameters	Range	Initial Parameters	Optimized Parameters
learning rate	0.001~0.01	0.01	0.0042
number of iterations	10~50	25	35
batch size	16~256	64	34
kernel size of convolutional layer 1	1~16	5	10
kernel number of convolutional layer 1	1~20	20	20
kernel size of convolutional layer 2	1~16	10	6
kernel number of convolutional layer2	1~20	20	20
neuron in fully connected layer 1	1~50	30	25
neuron in fully connected layer 2	1~50	10	39

## Data Availability

The data that support the findings of this study are not publicly available due to the confidentiality requirements of one ongoing project.

## Abbreviations

$a_{j}^{l}$	the output value	SSA	Sparrow Search Algorithm
$b_{j}^{l}$	the bias	ISSA	Improved Sparrow Search Algorithm
*d*	the distance	DGA	Dissolved Gas Analysis
*down*(.)	the pooling function	ELM	Extreme Learning Machines
*f_i_*	the fitness value	ANN	Artificial Neural Networks
*K*	the step size coefficient	SVM	Support Vector Machines
*M*	the window length	ES	Expert Systems
*M_j_^l^*	the selected set of input feature maps	ApEn	Approximate Entropy
*N*	size of the input data	MApEn	Multi-scale Approximate Entropy
*R*	the threshold	PSO	Particle Swarm Optimization
t	the iterations	GWO	Grey Wolf Optimizer
*Τ*	the time scale	GSA	Gravitational Search Algorithm
u(i)	he original signal	AVOA	African Vultures Optimization Algorithm
$X_{gauss}^{t}$	the Gaussian best solution	ReLU	Rectified Linear Unit
*X*	the original data matrix	NT	Normal type
*x*(i)	vectors with a dimension of m	HD	High-energy discharge
$y_{j}^{\left(\tau \right)}$	the coarse-grained process	LD	Low-energy discharge
$Z_{I}^{K}$	chaotic sequences	HO	High-temperature overheating
*β*_j_^l^	the multiplicative residua	ITO	Intermediate-temperature overheating
*β*	step size control parameter	ILO	Intermediate to low-temperature overheating
$\omega_{m}$	non-linearly decreasing weight	PD	Partial discharge
BPNN	Backpropagation Neural Network		
